# In-hospital evaluation of an app-based seizure detection system in dogs: timely detection of generalized tonic–clonic seizures

**DOI:** 10.3389/fvets.2025.1558274

**Published:** 2025-04-29

**Authors:** Junya Hirashima, Miyoko Saito, Daisuke Hasegawa, Rikako Asada, Masato Kitagawa, Daisuke Ito, Shinichi Kanazono, Koichi Fujiwara

**Affiliations:** ^1^Laboratory of Small Animal Surgery (Neurology), School of Veterinary Medicine, Azabu University, Sagamihara, Japan; ^2^Joint Department of Veterinary Medicine, Faculty of Applied Biological Sciences, Gifu University, Gifu, Japan; ^3^Laboratory of Veterinary Radiology, Nippon Veterinary and Life Science University, Musashino, Japan; ^4^Laboratory of Veterinary Neurology, School of Veterinary Medicine, Nihon University, Fujisawa, Japan; ^5^Neurology and Neurosurgery Service, Veterinary Specialists and Emergency Center, Kawaguchi, Japan; ^6^Department of Material Process Engineering, Nagoya University, Nagoya, Japan

**Keywords:** dog, epilepsy, seizure detection, accelerometer, wearable device

## Abstract

The seizure detection system (SDS) is a wearable device developed by us to detect generalized tonic–clonic seizures (GTCSs) in dogs with epilepsy. In our previous study, a feasibility test was conducted for the SDS, which demonstrated its ability to correctly identify three GTCSs in one dog. To enhance user accessibility and facilitate real-time monitoring of epileptic seizures in dogs, we integrated the system into a smartphone application. This study aimed to evaluate the performance of the app-based SDS in a clinical setting involving a larger number of dogs. Initially, the app-based SDS was tested on a laboratory dog with no history of seizures, and a drug-induced GTCS was accurately detected. Subsequently, an in-hospital evaluation was conducted. A total of 12 dogs were included, comprising 10 dogs with epilepsy, either hospitalized or temporarily housed at participating veterinary hospitals, and two laboratory dogs with epilepsy. In total, 34 GTCSs occurred in four of the 12 dogs, and the app-based SDS correctly detected 25 of the 34 GTCSs. Including the preliminary test results, the overall sensitivity of the app-based SDS was 74.3% (26 out of 35 GTCSs). Two false positives were observed in both in one dog. The false-positive rate and positive predictive value of the app-based SDS for detection of GTCS were 0.018 events/day and 92.6%, respectively. The median detection latency from the onset of a GTCS was 11 s. This study demonstrates that the app-based SDS is effective for detecting GTCSs in hospitalized dogs in clinical settings.

## 1 Introduction

Epilepsy is a chronic neurological disorder characterized by recurrent epileptic seizures and is a prevalent condition in dogs. Due to the unpredictable nature of spontaneous seizures, several studies have reported that owners of dogs with epilepsy experience significant stress, as the anxiety of sudden seizures severely impacts their quality of life (1–5). For veterinarians, prompt therapeutic intervention during seizures is important to prevent serious outcomes, such as status epilepticus, which is associated with high mortality (6). Continuous monitoring of these patients is challenging, both for most owners and the veterinary team during hospitalization. To resolve this, a system to detect and notify owners and clinicians about seizure episodes is warranted.

Various methods for detecting or predicting epileptic seizures have been investigated in humans, including electroencephalograms, electrocardiograms, accelerometers, video detection systems, and heart rate variability (7–13).

However, in veterinary medicine, only a few studies have focused on seizure detection, including our previous study (14, 15). We developed a preliminary seizure detection system (SDS) using a unique algorithm and acceleration data to detect generalized tonic-clinic seizures (GTCSs) in dogs (15). This GTCS detection method has been patented (16). In a previous study, we conducted a feasibility test for our preliminary SDS in three dogs with epilepsy, successfully detecting all three GTCSs in one dog (15). During the further refinement and validation process of our preliminary SDS, we developed a smartphone app-based SDS (named “Epi-Moni,” short for epilepsy-monitoring) that integrates the detection algorithm. This study aimed to evaluate the performance of our app-based SDS in a clinical setting with a large cohort of dogs. A preliminary experiment was conducted with a laboratory dog to confirm that the app-based SDS functioned properly. We then proceeded to evaluate the performance of the app-based SDS in a clinical setting.

## 2 Materials and methods

### 2.1 Animals

#### 2.1.1 The preliminary experiment

A healthy laboratory dog maintained at Azabu University was used in a preliminary experiment. The experimental protocol was approved by the Ethics Committee of the Azabu University, Japan (approval number 201007–4).

#### 2.1.2 In-hospital evaluation

This study was conducted at the Azabu University Veterinary Teaching Hospital, the Veterinary Medical Teaching Hospital of Nippon Veterinary and Life Science University, the Nihon University Animal Medical Center, and the Veterinary Specialist Emergency Center in Japan. The study period was from April 2020 to August 2021. The inclusion criteria were (1) dogs that had GTCSs as one of their seizure types, (2) dogs that accepted wearing a jacket equipped with the SDS, and (3) dogs hospitalized in the participating hospitals or temporarily housed in those hospitals during the consultation. Two laboratory dogs with epilepsy from Azabu University also participated in this study. As the primary goal of the study was to evaluate the performance of the app-based SDS, dogs with GTCSs were included regardless of the underlying causes of epilepsy. The study protocol was approved by the ethics committee of Azabu University, Japan (approval number 200330–1).

### 2.2 The app-based seizure detection system

**Figure 1:**
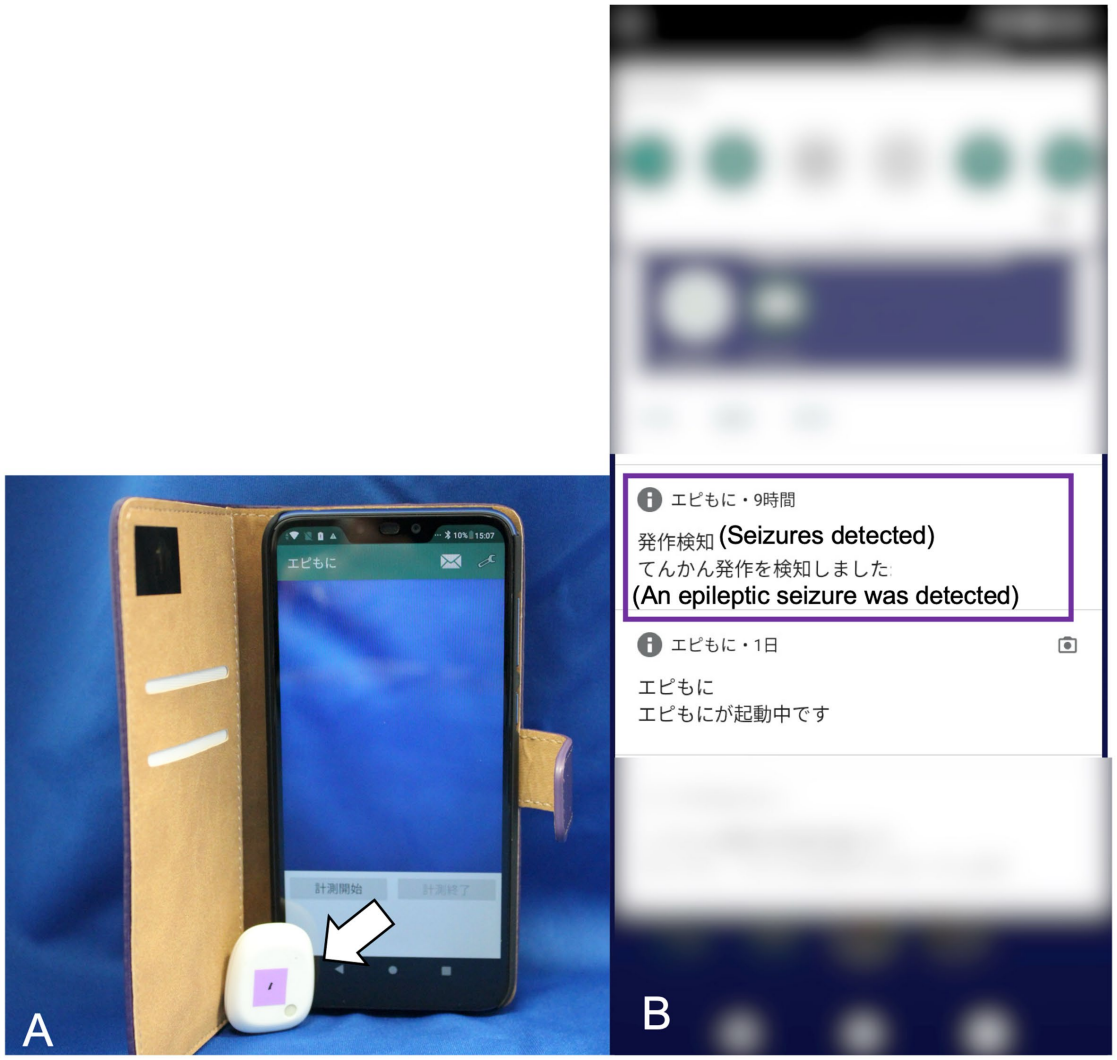
The app-based seizure detection system. The 3-axis accelerometer (indicated by the white arrow) has dimensions of 28 mm in width, 33 mm in height, and 10 mm in depth, with a weight of 7 g **(A)**. The smartphone display showing the seizure onset notification in Japanese (highlighted by the purple square line) **(B)**.

**Figure 2:**
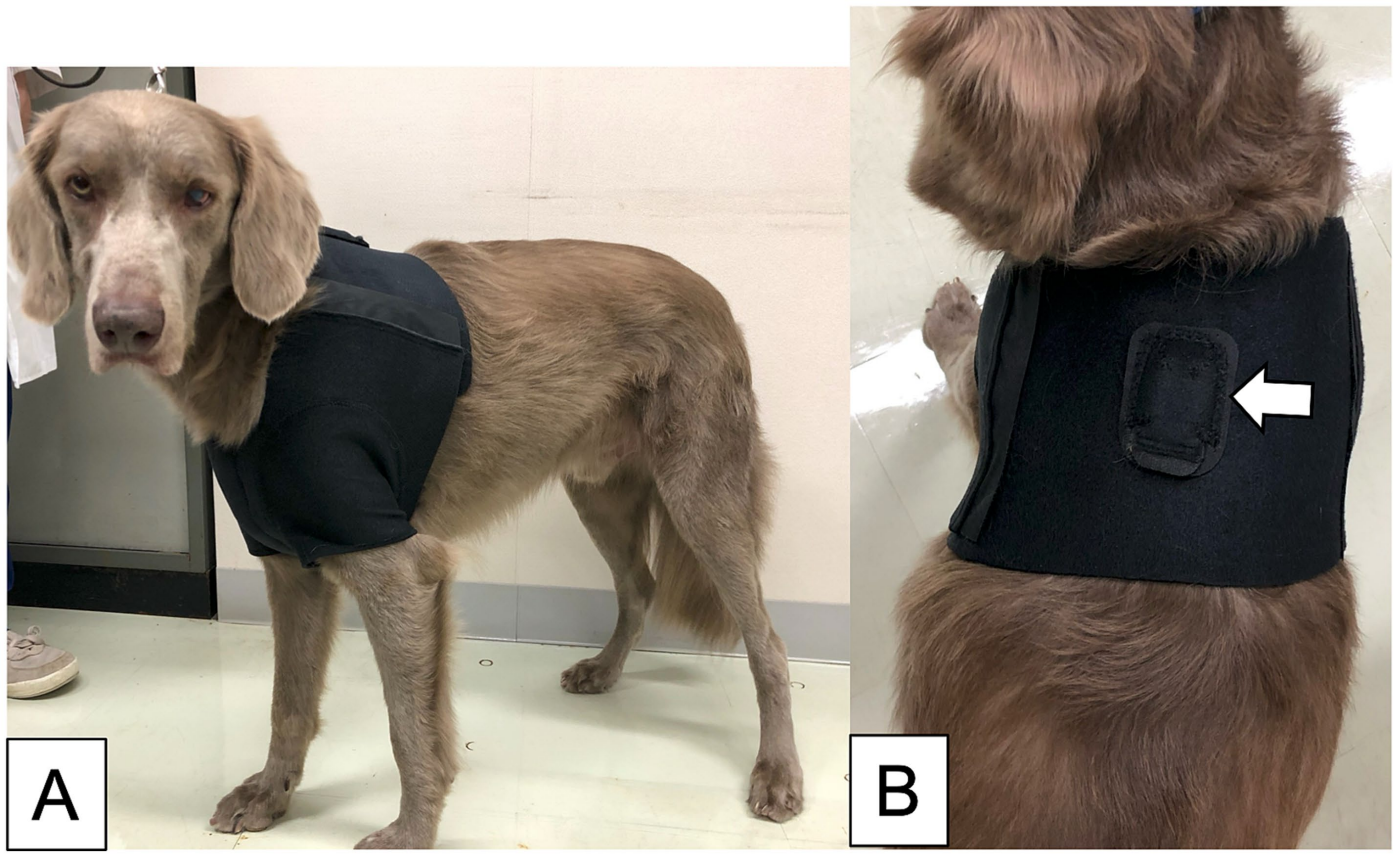
The jacket was made for this study. The dog wearing the size L jacket **(A)**. The dorsal view of the jacket, including the pocket for inserting the accelerometer (indicated by the white arrow) **(B)**.

The prototype of our SDS used in a previous study consisted of a three-axis accelerometer and a detection device that implemented the algorithm for the prior feasibility test (15). However, because the prototype device required a LAN cable to connect to a PC that ran the detection algorithm, its use was restricted to certain locations (15). To overcome this limitation, a new version of the SDS was developed for improved functionality. This version consisted of a three-axis accelerometer (MetaMotionR, MbientLAB) and a smartphone (Google Pixel 4a, Google) running a custom app that implements the developing algorithm (Figure 1A). The accelerometer and smartphone were connected via Bluetooth Low Energy (BLE). The Bluetooth connection had a distance limit of approximately 10 meters.The seizure detection algorithm calculates the Mahalanobis distance between the acceleration data of a test dataset and pre-generated reference datasets, which include datasets for GTCSs and daily activities. Details of the data processing in the detection algorithm are described in our previous study (15). When a GTCS was detected, the app-based SDS notified the smartphone monitor of seizure onset (Figure 1B). The sample rate of the accelerometer was 50 Hz with a range of ±8 g. According to the manufacturer, the battery life of the accelerometer ranged from 1 to 14 days. We used 2 accelerometers per case, alternating them daily to ensure continuous monitoring. Dogs wore specially designed jackets with pockets to house the accelerometer (Figures 2A,B). The jacket positioned the accelerometer in the interscapular region of the dog. The X-axis was in the craniocaudal direction, the Y-axis in the lateral direction, and the Z-axis in the dorsoventral direction. The jacket was available in four size ranges (XS, S, M, and L), with a suitable size selected for each dog.

### 2.3 Procedures

#### 2.3.1 The preliminary experiment

A GTCS was induced in one laboratory dog by intravenous administration of bemegride (Medibal; Mitsubishi Tanabe Pharma Corporation, Osaka, Japan), a drug previously used in our study to provoke GTCSs (15). The app-based SDS was activated prior to Bemegride administration. A dose of 20 mg Bemegride was administered intravenously over 30 s, inducing the GTCS. Following the cessation of the drug-induced GTCS, diazepam (0.5 mg/kg) and phenobarbital (3 mg/kg) were administered intravenously to prevent further seizures. Seizure detection was stopped once postictal signs such as mild ataxia resolved. The entire procedure was recorded using a web camera, and the smartphone and web camera were synchronized before the experiment.

After the experiment, the SDS data stored in the smartphone application were analyzed to confirm whether it had detected the drug-induced GTCS or not. If the app-based SDS detected the drug-induced GTCS, the detection latency, defined as the delay between seizure onset and detection by the app-based SDS, was measured using the recorded video. As a GTCS typically occurs secondary to a focal epileptic seizure, the detection latency measurement began when a focal epileptic seizure evolves into a GTCS. If a GTCS did not originate from a focal epileptic seizure, the detection latency was defined as from seizure onset. This experiment was conducted only once.

#### 2.3.2 In-hospital evaluation

**Figure 3:**
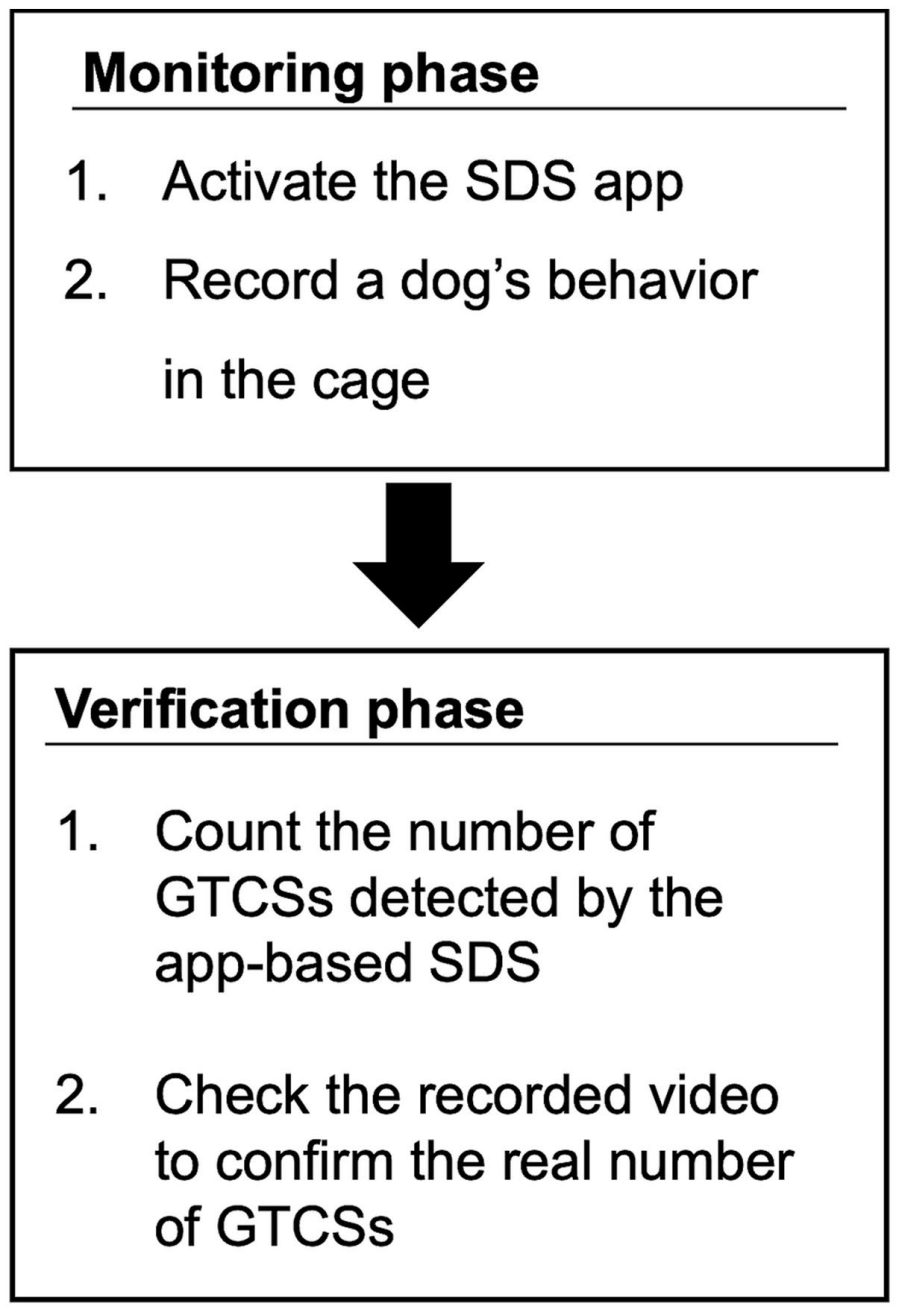
Flowchart of the procedure.

In this study, the evaluation procedure of the app-based SDS consisted of two phases: a monitoring phase and a verification phase. The flowchart of these phases is illustrated in Figure 3.

##### 2.3.2.1 Monitoring phase

**Figure 4:**
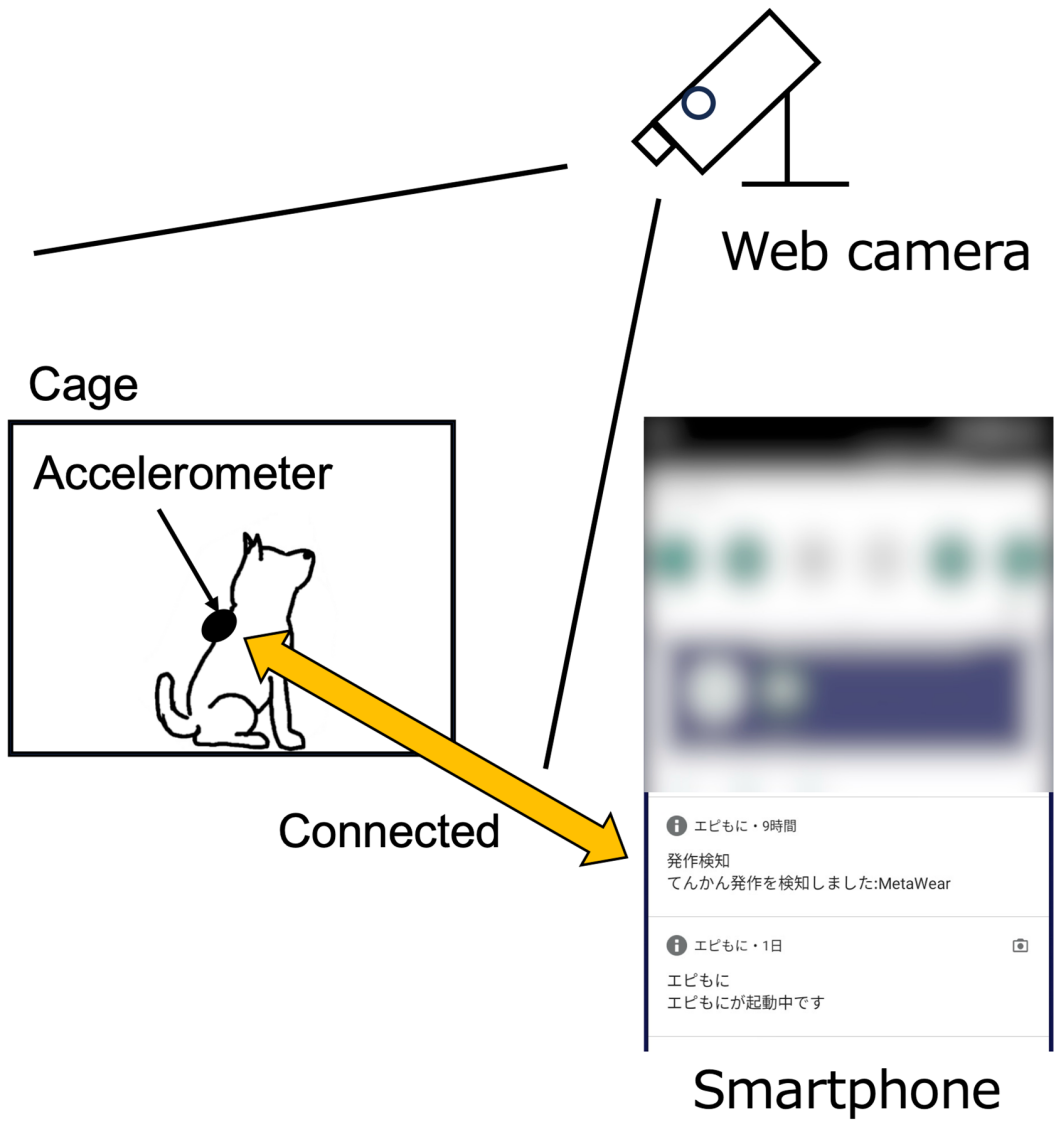
Schematic diagram of the experimental setting. The web camera was positioned to allow clear observation of the dog’s entire body.

The dog wore a jacket with an accelerometer, and the SDS app was launched (Figure 4). The dog was kept in a cage suitable for its body size, and the smartphone was placed close to the cage to minimize communication errors between the smartphone and the accelerometer. A web camera was used to record the dog’s behavior in the cage, including visual identification of GTCS episodes. A video camera was used to record the laboratory dogs. The time on the smartphone and the web or video camera were synchronized before the start of this study. The app-based SDS operated for up to 5 days in hospitalized dogs, depending on the storage capacity of the web camera, or until temporarily held dogs were returned to their owners. In two laboratory dogs with epilepsy, the app-based SDS was tested daily for as long as possible, except during the extraction of stored video data from the camera.

##### 2.3.2.2 Verification phase

After the monitoring phase, the SDS data stored in the smartphone app and the video recorded by the web or video camera were extracted. The extracted SDS and camera-based visual data were then analyzed to assess the detection rate, accuracy, and timing of SDS-based GTCS in comparison with video-based seizure data. In analyzing the video-based datasets, given the considerable variation in tonic–clonic seizures in dogs, any seizures exhibiting both tonic and clonic phases were classified as tonic–clonic. Detection latency was measured in the same manner as in the preliminary experiment. Finally, the entire recorded video was reviewed to verify whether the dog had an undetected GTCS. Periods in which the dog’s behavior could not be assessed using the recorded video were excluded from the analysis.

One author (JH) extracted the data logs from the smartphone, recorded the video from the web or camera, and analyzed the data logs as described above.

If a participant received anti-seizure medications, they continued their routine medication regimen during the study. Moreover, in hospitalized dogs, additional anti-seizure medications were administered as needed to prevent seizures. Occurrence of GTCSs is necessary for this study; however, we prioritized seizure prevention as clinicians.

When the clinical staff observed seizure onset in a participating dog while the app-based SDS was running, therapeutic intervention was performed immediately, regardless of whether the app-based SDS detected the GTCS. Since interventions, such as restraining the dog during seizures, could potentially influence the accurate evaluation of the app-based SDS, such GTCSs were excluded from the evaluation when these interventions were visible in the recorded video. However, if the app-based SDS detected the GTCS prior to human intervention, the GTCS was included in the analysis. Generalized tonic–clonic seizures were also excluded if the dog’s jacket was found to be detached or improperly worn during or before seizure onset. Seizure detections occurring more than 5 min after seizure onset were considered a detection failure.

False positives: A false positive, defined as the detection of a GTCS when no seizure occurred, was excluded if the dog’s jacket was detached or not properly worn at the time of detection.

### 2.4 Outcome measures of in-hospital evaluation

The outcome measures of this study included (1) sensitivity, (2) false positive rate, (3) positive predictive value, and (4) detection latency. The definitions of each term are as follows: Sensitivity was defined as the ratio of GTCS detected accurately by the app-based SDS to the total number of GTCSs in dogs. The false positive rate was calculated as the ratio of false positives to the total number of app-based SDS running days. The positive predictive value was the ratio of true GTCSs detected by the app-based SDS to the total number of GTCSs detected by the app-based SDS (including false positives).

The secondary outcome was whether focal epileptic seizures that did not evolve into GTCSs were detected by the app-based SDS.

### 2.5 Statistical analysis

This study used a descriptive analysis. The interquartile range was calculated using JMP Pro 17 software (SAS Institute Japan Ltd., Japan).

## 3 Results

### 3.1 Animals

#### 3.1.1 Preliminary examination

A 156-month-old intact male Beagle with no history of seizures was used in the preliminary examination. The body weight of the dog was 10.1 kg.

#### 3.1.2 In-hospital evaluation

Twelve dogs were included in the in-hospital evaluation: 10 client-owned dogs with epilepsy and two laboratory dogs with epilepsy. The cohort comprised three Beagles and one each of a Shetland Sheepdog, Weimaraner, mixed-breed dog, Toy poodle, Cavalier King Charles Spaniel, Boston Terrier, Shiba dog, Miniature Dachshund, and Maltese. There were five intact males, four spayed females, and three neutered males. The median age and body weight of the dogs are 77.5 months (range: 18–145 months) and 9.65 kg (range: 2.0–32.6 kg), respectively.

### 3.2 Running time of the seizure detection system

#### 3.2.1 The preliminary examination

The running time of the app-based SDS was 24 min.

#### 3.2.2 In-hospital evaluation

The total running time of the app-based SDS for all dogs was 160,380 min (111 days). The median running time of the app-based SDS for each dog was 1,705 min (IQR: 136–11,563 min, range: 28–109,084 min). There were no periods in which the behavior of the dogs could not be assessed.

### 3.3 Outcomes

#### 3.3.1 The preliminary examination

The app-based SDS accurately detected drug-induced GTCS in a laboratory dog. No false positive results were observed during the experiment. The detection latency was measured at 15 s.

#### 3.3.2 In-hospital evaluation

The performance results of the app-based SDS for each dog are presented in Table 1. A total of 37 spontaneous GTCSs occurred in five dogs (Dogs B, G, H, I, and J). Dog J experienced two GTCSs, both of which received immediate intervention from the clinical staff before the app-based SDS detected those GTCSs. Consequently, the GTCSs in Dog J were excluded from the evaluation. Although Dog G had five GTCSs, one episode was excluded because the jacket was not worn correctly during the GTCS.

**Table 1:** The result of seizure detection in each dog.

	Number of GTCSs	Number of GTCSs that the SDS detected	Number of GTCSs excluded from evaluation	Number of false positives	Sensitivity (%)	Running time of the app-based seizure detection system (minutes)
Dog A (Laboratory Beagle)	0	0	0	0	Not calculated	2,392
Dog B (Laboratory Beagle)	24	22	0	2	91.7 [22/24]	109,084
Dog C (Shetland Sheepdog)	0	0	0	0	Not calculated	211
Dog D (Weimaraner)	0	0	0	0	Not calculated	790
Dog E (mix-breed dog)	0	0	0	0	Not calculated	101
Dog F (Toy poodle)	0	0	0	0	Not calculated	28
Dog G (Cavalier King Charles Spaniel)	5	3	1	0	75.0 [3/4]	3,920
Dog H (Boston Terrier)	5	0	0	0	0 [0/5]	23,040
Dog I (Beagle)	1	0	0	0	0 [0/1]	13,285
Dog J (Shiba dog)	2	0	2	0	Not calculated	6,399
Dog K (Miniature Dachshund)	0	0	0	0	Not calculated	1,018
Dog L (Maltese)	0	0	0	0	Not calculated	112

After excluding these three episodes, a total of 34 GTCSs observed in four dogs (Dogs B, G, H, and I) were evaluated. The app-based SDS detected 25 out of the 34 GTCSs, yielding an overall sensitivity of 73.5%. The median sensitivity for each dog was 37.5% (IQR: 0–87.5%, range: 0–91.7%). One detected GTCS (Dog G) is shown in Supplementary Video 1, and one undetected GTCS (Dog H) is shown in Supplementary Video 2.

Three false positives were recorded in Dog B, while no false positives were observed in the other 11 dogs. One of the three false positives was excluded from the evaluation because the jacket was not worn correctly at the time of the episode. The remaining false positives occurred when the dog slipped frequently in a cage after recovering from anesthesia for an MRI scan to determine the cause of epilepsy and when the dog struggled during an injection of an anti-seizure drug. Thus, the false-positive rate was 0.018 per day.

The positive predictive value was calculated for all dogs, with the app-based SDS detecting 27 GTCSs, including two false positives, resulting in a positive predictive value of 92.6% (25 of 27 detections).

The median detection latency, measured from the onset of the GTCSs, was 11 s (IQR: 9.5–14.0 s, range: 6–75 s).

Additionally, two of the 12 dogs experienced 35 focal epileptic seizures that did not evolve into GTCSs. These focal epileptic seizures consisted of facial twitching. The app-based SDS did not detect any of these seizures.

#### 3.3.3 The overall sensitivity

One drug-induced GTCS was detected during the preliminary examination, and 25 of the 34 spontaneous GTCSs were detected during the in-hospital evaluation. Therefore, the overall sensitivity of the app-based SDS was 74.3% (26 of 35 GTCSs).

## 4 Discussion

We evaluated the performance of our SDS using a previously developed detection algorithm (15). In humans, the sensitivity of epileptic seizure detection with accelerometers ranges from 79 to 91% (7–9). The overall sensitivity in our study was 74.3%, which is comparable to that obtained in human studies. In veterinary medicine, a study investigating the sensitivity of accelerators for detecting generalized seizures in 136 seizures from 19 dogs reported a sensitivity of 22.1% (14). Differences between previous and present studies, such as data processing methods, accelerometer sampling rates, and placement of the accelerometer in dogs, may explain the differences in sensitivity between the two studies. Given that the sample size in our study was smaller than that in the previous study (14), further evaluations are warranted to better assess the performance of the SDS.

Regarding false positives, only two instances occurred in one of the 12 dogs, yielding a false-positive rate of 0.018 per day. Because human intervention occurred during the two false-positive events, the app-based SDS was deemed less likely to produce false positives when the dog was left alone. We did not exclude false positives that occurred during human intervention, as we aimed to confirm the circumstances under which false positives occurred. Although the overall sensitivity of our algorithm was 74.3%, the false positive rate was considerably low, while the positive predictive value was high. Minimizing false positives is essential for accurate seizure detection. Hence, we consider the specificity of the app-based SDS to be reliable. We presume that the app-based SDS has significant potential as an effective seizure detection device.

Timely detection of seizures is critical for effective intervention. Due to the specifications of our seizure detection algorithm, the app-based SDS required at least 9 s from a GTCS onset to detect it (15), with a median detection latency of 11 s in this study. Our algorithm both detects and determines seizures simultaneously (15). It divides the recorded acceleration data into epochs of 9 s each. These 9-s epochs are created repeatedly, with the beginning of each epoch shifted by 1 s. Our algorithm immediately determines whether a seizure has occurred or not for each of these continuously created 9-s epochs. Thus, in our study, GTCS was detected with a median latency of 11 s after onsets. This indicates that the performance of the app-based SDS is comparable to that of the original version, as both systems utilize the same detection algorithm. The longest detection latency recorded in this study was 75 s. Status epilepticus, which can be life-threatening, is clinically defined as “*continuous epileptic seizures lasting more than 5 min*” (17). If generalized seizures could be detected and caregivers alerted within an average of 11 s, it would provide an opportunity for timely medical interventions, such as intranasal midazolam administration (18), potentially preventing progression to status epilepticus. This highlights an additional clinical benefit of the app-based SDS: the timely detection of GTCSs.

The app-based SDS did not detect GTCSs in two dogs. Although GTCSs involve both tonic and clonic seizures, the combined patterns of tonic and clonic movement vary between dogs (19). Some dogs exhibit predominantly tonic seizures, while others have predominantly clonic seizures. Therefore, even if the seizure is classified as a GTCS, there may be a combination of tonic and clonic movements that cannot be accurately detected by our algorithm. Moreover, the variability and speed of recorded acceleration data may be important for our system’s GTCS detection. Our algorithm calculates the coefficient of variation (CV) from the recorded acceleration data (15), with a higher CV indicating greater variability. Our other study (unpublished data) showed that detected GTCSs had higher CV and faster acceleration than undetected ones. Therefore, as shown in the supplementary movies, our system appeared to identify seizures as GTCS when there were intense movements with sudden changes in seizure activity. However, elucidating why some GTCS are detected and others are not is beyond the scope of this study. To further investigate the differences in characteristics between detected and undetected GTCSs, a larger sample size with more GTCS cases is needed.

The accelerometer was placed in the interscapular region of the dog for the SDS. In our previous study, we found that displacement of the accelerometer from its proper position caused false positives (15). As a result, we specifically designed a jacket to secure the accelerometer, minimizing any rotation or displacement. Therefore, proper wearing of jackets is essential for the SDS’s accuracy. In our study, no issues were encountered with any of the dogs wearing jackets. We presumed that if the accelerometer were attached to a collar, it could rotate significantly, which might hinder accurate seizure detection. Moreover, we believe that the interscapular region provides protection for the accelerometer, preventing damage during seizures. Therefore, we determined that the interscapular region was a suitable position for our SDS.

Although focal epileptic seizures characterized by facial twitching occurred in two dogs during the observation period, our algorithm was designed solely to detect GTCSs, which was our primary objective in developing the device. We prioritized GTCS detection because it is the most common seizure type in dogs (6, 20, 21) and is most frequently associated with status epilepticus. We used the acceleration data obtained from GTCSs as training data for the algorithm (15). As expected, the app-based SDS did not detect focal epileptic seizures. Given the variety of seizure types in canine epilepsy (17), a seizure detection algorithm tailored for each specific seizure type may be required in the future.

This study had several limitations. Although 12 dogs participated in the study, only four showed spontaneous GTCSs during the monitoring phase. Moreover, the most of the seizures occurred in one laboratory dog. To accurately assess the performance of the app-based SDS, a larger number of dogs with GTCSs is required. Additionally, we confirmed GTCSs only by the recorded video and no supplementary electroencephalographic (EEG) data to support our findings. This limitation may have resulted in the underreporting of seizure episodes, although confirmation via EEG was not a primary aim of this study. A comparison of the SDS and video data was conducted by a single author (JH). As a result, inter-observer agreement in the visual identification of the video-based dataset was not assessed, which could introduce bias in the counted number of seizures. This study used an app-based SDS for dogs hospitalized and kept in cages. For future advancements, evaluating the app-based SDS in dogs in different situations, such as home settings, is recommended.

This study revealed that the overall sensitivity of the app-based SDS was 74.3%. The occurrence of false-positive findings was very low (0.018 per day). The median detection latency was 11 s, which may be sufficient to initiate therapeutic interventions to prevent status epilepticus. Although a higher sensitivity would be ideal for optimal seizure detection, the low false positive rate and short detection latency suggest that SDS has considerable potential for clinical applications for detection of GTCS in hospitalized dogs.

## Data Availability

The original contributions presented in the study are included in the article/Supplementary material, further inquiries can be directed to the corresponding author.

## Supplementary material

Supplementary Video 1 A detected generalized tonic-clonic seizure with the app-based Seizure Detection System, showing intense movements and sudden changes in seizure activity.

Supplementary Video 2 An undetected generalized tonic-clonic seizure with the app-based Seizure Detection System, which appears to have less intense movements and fewer sudden changes in seizure activity compared to Video 1.
